# Decreased quality of life in Duchenne muscular disease patients related to functional neurological and cardiac impairment

**DOI:** 10.3389/fneur.2024.1360385

**Published:** 2024-02-08

**Authors:** Lenka Juříková, Lucia Masárová, Roman Panovský, Martin Pešl, Kamila Žondra Revendová, Ondřej Volný, Věra Feitová, Tomaš Holeček, Vladimír Kincl, Pavlína Danhofer, Stanislav Voháňka, Jana Haberlová, Karolína Podolská

**Affiliations:** ^1^Department of Paediatric Neurology, Faculty of Medicine of Masaryk University, University Hospital Brno, Brno, Czechia; ^2^International Clinical Research Center, St. Anne’s University Hospital, Faculty of Medicine, Masaryk University, Brno, Czechia; ^3^1st Department of Internal Medicine-Cardio-angiology, Faculty of Medicine, St. Anne’s University Hospital, Brno, Czechia; ^4^Department of Biology, Faculty of Medicine, Masaryk University, Brno, Czechia; ^5^Department of Neurology, University Hospital Ostrava, Brno, Czechia; ^6^Centre for Clinical Neurosciences, Faculty of Medicine, University Ostrava, Ostrava, Czechia; ^7^Department of Medical Imaging, St. Anne’s University Hospital, Brno, Czechia; ^8^Department of Biomedical Engineering, University of Technology, Brno, Czechia; ^9^Department of Neurology, Faculty of Medicine, University Hospital Brno, Masaryk University, Brno, Czechia; ^10^Department of Paediatric Neurology, 2nd Faculty of Medicine, Charles University and Motol University Hospital, Prague, Czechia

**Keywords:** quality of life, Duchenne muscular dystrophy, cardiac magnetic resonance, neurological status, cardiac impairment

## Abstract

In this prospective study involving 37 Duchenne muscular dystrophy (DMD) patients aged 8–18 years and older, we examined the impact of neurological and cardiac factors on quality of life (QoL). Our findings revealed a negative correlation between upper limb movement and overall mobility, self-service, and usual activities. Ambulatory and non-ambulatory DMD patients showed significant differences in mobility-related parameters. Cardiac evaluations demonstrated associations between mitral annular plane systolic excursion (MAPSE) and mobility-related aspects. The PEDSQL 3.0 neuromuscular model questionnaire further highlighted age-related and movement-related correlations with QoL. The loss of ambulatory status and reduced upper limb movement were negatively associated with QoL, while upper limb movement positively correlated with septal MAPSE. However, no significant associations were found between MAPSE and anxiety/depression. These findings underscore the multifaceted impact of DMD on QoL and emphasize the importance of considering both neurological and cardiac factors in comprehensive patient care.

## Introduction

The most common X-recessive inherited progressive muscular disease is Duchenne muscular dystrophy (DMD). It affects approximately 1:5000 living boys (1). The first symptoms of DMD manifest at the age of two years because of the absence of dystrophin protein, leading to gradual weakness and muscle injury (2).

DMD is still causally incurable (3). Due to the advances in medical care and symptomatic therapy, the life expectancy of DMD patients has been prolonged and their quality of life (QoL) has been improving (4, 5). Today, DMD patients live up to 30 or 40 years (6). It is well-known that DMD influences the QoL not only for patients, but also for their families (7–11).

According to the World Health Organization, QoL is defined as a person’s perception of their position in life including the culture and values in the system in which the person lives, correlating to a person’s goals, expectations, standards, and concerns. It is a complex of physical health, psychological status, level of independence, social relationships, personal beliefs, and a person’s relationship with various features of the environment (12). Published reports about the QoL in DMD patients are not uniform. Some of the previous studies reported a decline only in physical functions compared to healthy controls, while the others show that a whole spectrum of QoL parameters were decreased to age-matched controls (7, 13–15). The results may also have been influenced by self/proxy reports (by the parents/caregivers) (16–22) or by the help of pediatricians (14).

The Pediatric Quality of Life (PEDSQL) 3.0 Neuromuscular Module questionnaire among children/adolescents, EQ-5D, and Individualized Neuromuscular QoL questionnaire for adults are the most commonly used questionnaires in the published studies (23–26).

To date, only one study describing the QoL in DMD patients related to respiratory and cardiac functions including left ventricular ejection fraction (LVEF) evaluated by echocardiography and the presence or absence of electrocardiogram (ECG) abnormalities or cardiomyopathy has been published (27).

To our knowledge, there has been no prospective study describing the QoL of all-aged DMD patients in correlation with a complex evaluation firstly of neurological clinical status and cardiac impairment using cardiac magnetic resonance (CMR), and secondly of the influence of cardiac therapy or corticosteroid therapy, body mass index (BMI), and anxiety/sadness.

### Objectives

The main purpose of this prospective study was to investigate the QoL concerning neurological clinical status and cardiac impairment using CMR, and the influence of actual therapy, BMI, and anxiety/sadness in DMD patients based on a completed questionnaire.

## Methods

### Participants

DMD patients 8–18 years and older born in the Czech Republic were consecutively included between 01/2022 to 09/2023 in close cooperation with EndDuchenne (a patient advocacy group). All DMD patients were diagnosed based on their clinical symptoms and/or elevated creatine kinase, and confirmed by genetic testing. The most common genetic mutation was the deletion of 44–63 exons in 65% of our cohort.

This study was performed in accordance with the Declaration of Helsinki (2000) of the World Medical Association and was approved by the Institutional Ethics Committee (University Hospital Brno, reference number 20130410–03). All DMD patients or their parents (for patients younger than 18 years) signed the informed consent.

Every patient was examined by an experienced pediatric neurologist and also an experienced cardiologist including ECG and ECG Holter, and the following pieces of information were collected: demographic data (age, weight, height), current pharmacotherapy (corticosteroid therapy, beta-blockers, angiotensin converting enzyme inhibitors (ACEI), sartan-losartan (generic name of sartan), diuretics), comorbidities (hypertension, diabetes mellitus, chronic renal insufficiency), an actual feeling of dyspnoea and palpitations.

All DMD patients were monitored for respiratory status such as a need of nocturnal non-invasive positive pressure ventilation (NIPPV). Six DMD patients of our cohort use nocturnal NIPPV. Neurological clinical status included the following information - ambulatory/non-ambulatory, self-sitting or sitting requiring help, and the presence of scoliosis including a history of scoliosis surgery. Upper limb movement was assessed in 5 various degrees: preserved mobility, moderately limited mobility, limited mobility, very limited mobility, and work with touchpad/mouse.

DMD patients were divided into three groups based on age: 8–12 years, 13–18 years, and adult patients. All of them were asked to complete questionnaires for muscular dystrophy. The younger patients completed the PEDSQL 3.0 neuromuscular model questionnaire, and the older ones EQ-5D. Due to possible differences between self and proxy reports, we employed a PEDSQL 3.0 neuromuscular model questionnaire completed by DMD patients and their parents. All DMD patients and parents were instructed by an experienced pediatric neurologist how to complete the questionnaire in electronic version. None of the DMD patients were taking any psychotropic medications or opioids when the questionnaire was administered.

### Questionnaires

#### PEDSQL 3.0 neuromuscular model questionnaire

PEDSQL is an instrument used to assess Health-Related QoL (HRQoL) in children and adolescents aged 2 to 18. It consists of both generic core and disease-specific modules. The PEDSQL 3.0 Neuromuscular Module questionnaire is a disease-specific module for measuring children’s QoL assessing their neuromuscular disease, communication difficulties, and family resources. This instrument is acknowledged as a validated health outcome measure for patients with neuromuscular diseases (28). It was validated for the Czech Republic (29) for the DMD patient cohort and was translated into the Czech language. Its electronic version can be requested through this web page ePROVIDE™ - Online Support for Clinical Outcome Assessments.^1^

#### EQ-5D questionnaire

EQ-5D questionnaire is a standardized measure of HRQoL developed by the EuroQol Group to provide a simple, generic questionnaire for use in clinical and economic appraisal and population health surveys. EQ-5D assesses health status in terms of five dimensions of health and is considered a ‘generic’ questionnaire because these dimensions are not specific to a specific patient group or health condition. EQ-5D can also be referred to as a patient-reported outcome measure (PROM) because patients can complete the questionnaire themselves to provide information about their current health status and how this changes over time (30). It was validated for the Czech population for patients with chronic pain (31) and can be found on the following web page: Available versions – EQ-5D (euroqol.org).

After a baseline clinical check-up and collection of all patient information, the DMD patients underwent a neurological examination. On the same day the patients were examined, the questionnaire in the electronic version was completed. After completing the questionnaire, DMD patients were examined by CMR within one week after their clinical examination by a neurologist.

Inclusion criteria for CMR: the absence of CMR contraindications such as an implanted pacemaker/defibrillator, cochlear implant, other ferromagnetic metal parts in the patient’s body, claustrophobia, etc.; the absence of contraindications for using contrast media such as severe renal insufficiency; the patient’s ability to co-operate during CMR examination; no known cardiovascular pathology apart from dystrophin cardiomyopathies.

All DMD patients underwent the CMR according to our previously published protocol (32) using a 1.5 T scanner (Ingenia, Philips Medical Systems, Best, The Netherlands) equipped with 5- and 32-element phased array receiver coils allowing for the use of parallel acquisition techniques in the supine position in repeated breath-hold. Four DMD patients underwent CMR examination without application of contrast agent.

#### CMR protocol

Functional imaging using balanced steady-state free precession cine sequences included four-chamber, two-chamber, and LV outflow tract long axis views, and a short axis stack from the cardiac base to the apex in the perpendicular plane to the LV long axis.

Late gadolinium enhancement (LGE) images in all long-axis views and the short-axis views were acquired 10 min after an intravenous bolus of 0.2 mmol/kg of the gadolinium-based contrast agent gadobutrol (Gadovist, Bayer-Schering Pharma, Germany) using an inversion-recovery turbo field echo sequence and, in case of doubt, also by phase-sensitive inversion recovery turbo field echo. Both 2-dimensional and 3-dimensional data acquisitions were performed in mid-diastole.

#### CMR analysis

The following parameters were evaluated: LVEF, end-diastolic/end-systolic volume (EDV/ESV), septal/lateral/average m*itral annular plane systolic excursion (MAPSE),* presence/absence of LGE. LV functional and morphological parameters were calculated from the short axis view stack using the summation-of-disc methods in accordance with recommendations for post-processing evaluation from the Society for Cardiovascular Magnetic Resonance (33). Septal and lateral MAPSE was measured as previously described (34, 35) by defining end-diastolic and end-systolic mitral annular planes on a long-axis four-chamber view. The average MAPSE was calculated as the mean of septal and lateral MAPSE.

LGE was defined as an area of visually identified contrast enhancement greater than the mean signal intensity of an adjacent area of the reference myocardium. LGE was not evaluated in four DMD patients who underwent the examination without application of contrast agent.

#### Statistical analysis

The data were analyzed using Stata Statistical Software Release 17 (StataCorp, College Station, TX). The continuous variables were reported as means and standard deviations (SDs) or medians and interquartile ranges (IQRs) according to the data distribution. The normality of data was evaluated by the Shapiro–Wilk test. Categorical variables were reported as counts and percentages. The Spearman correlation was used to assess the relationship between the components of the PEDSQL 3.0 neuromuscular model questionnaire and EQ-5D, age, BMI, upper limb movement, and CMR parameters. Similarly, the relationship between upper limb movement and CMR parameters was evaluated using the Spearman correlation. The Spearman’s rank correlation coefficient was interpreted according to Prion and Haerling (36). The Wilcoxon rank-sum (Mann–Whitney) test was used to assess differences in the components of the PEDSQL 3.0 neuromuscular model questionnaire and EQ-5D between the predefined subgroups (heart failure treatment, current corticosteroid therapy, the ability to walk, the ability to sit, the presence of scoliosis, the presence of LGE). The difference between the categories of the PEDSQL 3.0 neuromuscular model questionnaire filled in by the parent or by the patient was evaluated using the Wilcoxon signed-rank test. All tests were two-tailed, and *p* values <0.05 were considered statistically significant.

## Results

A total of 37 DMD patients (median age 16.8 years) were enrolled in the final analysis. The basic demographic data are shown in Table 1. The detailed information about current corticosteroid therapy is shown in Table 2. Results from both questionnaires are shown in Table 3.

**Table 1:** Basic demographic data.

Variable	
Age, median (IQR)	16.8 (13.9–20.7)
Weight, median (IQR)	50 (40–76)
Height, mean (SD)	1.55 (0.19)
Ambulatory patients (*n*, %)	19 (51.4%)
Self-sitting patients (*n*, %)	24 (64.9%)
Upper limbs movement, median (IQR)	4 (2–5)
Scoliosis (*n*, %)	19 (51.4%)
Surgery of scoliosis (*n*, %)	2 (5.4%)
Actual feeling of dyspnea (*n*, %)	2 (5.4%)
Actual feeling of palpitations (*n*, %)	0 (0%)
Arrhythmia (*n*, %)	0 (0%)
NIPPV (*n*, %)	6 (16,2%)
Diabetes mellitus (*n*, %)	0 (0%)
Chronic renal insufficiency (*n*, %)	0 (0%)
Corticosteroid therapy (*n*, %)	20 (54.1%)
ACEI/Sartans (*n*, %)	28 (75.7%)
LVEF, median (IQR)	64 (53–68)
EDV, median (IQR)	91 (70–119)
MAPSE septal	10.5 (9–12)
MAPSE lateral	11 (10–13)
MAPSE average	11 (10–11.5)
Presence of LGE	22 (66.7%)^*^

ACEI, angiotensin converting enzyme inhibitors; EDV, end-systolic volume; IQR, interquartil range; LGE, late gadolinium enhancement; LVEF, left ventricular ejection fraction; MAPSE, mitral annular plane systolic excursion; NIPPV, Non-invasive positive pressure ventilation; *n*, number, *p*, *p*-value, SD, standard deviation. ^*^Data only for 33 patients.

**Table 2:** Detailed information of corticosteroid therapy.

Corticosteroid therapy	DMD patients (*n*, %)
Currently	20 (54,1%)^*^
Never	14 (37%)
Previously	3 (8,1%)

DMD, Duchenne muscular dystrophy; *n*, number. ^*^One patient takes deflazacort.

**Table 3:** PEDSQL 3.0 neuromuscular model and EQ-5D results.

Scale	*N*	Median (IQR)
PEDSQL3.0 neuromuscular model questionnaire		
Child self-report		
Disease	19	19 (15–30)
Communication	19	6 (3–8)
Family	19	8 (6–11)
Parent report		
Disease	18	23.5 (17–34)
Communication	18	6.5 (4–9)
Family	18	8 (6–13)
EQ-5D		
Total mobility	36	3 (2–3)
Self-service	37	2 (2–3)
Usual activities	37	2 (2–3)
Pain	36	2 (1–2)
Anxiety	37	1 (1–2)
Good/bad days	37	7 (5–8)

IQR, interquartile range; *n*, number; PEDSQL, Pediatric quality of life questionnaire.

### EQ-5D questionnaire

DMD patients taking ACEI/sartans had better total mobility than DMD patients without therapy (*p* = 0.048).

There was a negative correlation between the movement of the upper limbs and total mobility (r_s_ = −0.619, *p* = 0.0001), self-service (r_s_ = −0.863, *p* < 0.001), and usual activities (r_s_ = −0.765, *p* < 0.001). On the contrary, movement of the upper limbs positively correlated with good/bad days (r_s_ = 0.515, *p* = 0.001). No correlation was found between the movement of the upper limbs, anxiety, and pain.

There was a statistically significant difference in total mobility (*p* < 0.001), self-service (*p* < 0.001), usual activities (*p* = 0.0001), and good and bad days (*p* = 0.0095) when comparing the ambulatory/non-ambulatory DMD patients (*p* < 0.001).

Similar results were found when comparing sitting/non-sitting DMD patients. There was a significant difference in total mobility (*p* < 0.001), self-service (*p* < 0.001), usual activities (*p* < 0.001), and good/bad days (*p* = 0.039) (see Table 4). No difference was found for anxiety and pain. DMD patients with or without scoliosis showed a statistical difference only on good/bad days (*p* = 0.011).

**Table 4:** QoL and neurological impairment in our DMD cohort.

Scale	*n*	Sitting	Non-sitting	*p*	Ambulatory	Non-ambulatory	*p*
PEDSQL 3.0 neuromuscular model							
Child self-report							
Disease	19	18.5 (14.5–28)	30 (28–36)	0.114	17 (14.5–23.5)	28 (28–31)	0.113
Communication	19	4 (3–8)	7 (6–9)	0.258	3.5 (2–7.5)	7 (4–8)	0.223
Family	19	8 (4–10.5)	7 (6–16)	0.935	7.5 (1–10)	8 (7–15)	0.392
Parent report							
Disease	18	18 (17–30)	35 (31–39)	0.071	17 (14–30)	31 (24–35)	0.097
Communication	18	6 (3–8)	9 (6–12)	0.201	7 (3–8)	6 (5–12)	0.425
Family	18	7 (6–16)	7 (6–16)	1.000	8 (7–15)	8 (2–10)	0.385
EQ-5D							
Total mobility	36	2 (2–3)	3 (3–3)	<0.001	2 (1–2)	3 (3–3)	<0.001
Self-service	37	2 (1–2)	3 (3–3)	<0.001	2 (1–2)	3 (2–3)	<0.001
Usual activities	37	2 (1–2)	3 (3–3)	<0.001	2 (1–2)	3 (2–3)	<0.001
Pain	36	2 (1.5–2)	2 (1–2)	0.517	2 (1–2)	2 (1–2)	1.000
Anxiety	37	1 (1–2)	1 (1–2)	0.578	1 (1–2)	1 (1–2)	0.864
Good/bad days	37	7 (5–8)	5 (4–7)	0.039	7.5 (6–8)	5 (4–7)	<0.001

DMD, Duchenne muscular dystrophy; QoL, quality of life; *n*, number; *p*, *p*-value; PEDSQL, Pediatric quality of life. Scales are reported as medians and IQRs.

When evaluating cardiac parameters, the following statistically significant correlations were found: a weak positive correlation between EDV and anxiety (r_s_ = 0.330, *p* = 0.046), a weak negative correlation between average MAPSE and total mobility (r_s_ = −0.386, *p* = 0.020), lateral MAPSE and total mobility (r_s_ = −0.344, *p* = 0.040), septal MAPSE and total mobility (r_s_ = −0.355, *p* = 0.034), and septal MAPSE and usual activities (r_s_ = −0.342, *p* = 0.039) (Table 5).

**Table 5:** Correlation between QoL and cardiac impairment using CMR.

Variable	*n*		LVEF	EDV	ESV	MAPSE average	MAPSE lateral	MAPSE septal
PEDSQL 3.0 neuromuscular model								
Child self-report								
Disease	19	r_s_	0.106	−0.044	0.047	0.041	0.102	0.002
		P	0.665	0.859	0.849	0.867	0.677	0.994
Communication	19	r_s_	0.257	−0.196	0.140	−0.115	−0.063	−0.040
		P	0.288	0.422	0.569	0.641	0.799	0.873
Family	19	r_s_	0.088	−0.256	−0.117	0.027	0.001	0.017
		P	0.721	0.290	0.634	0.913	0.999	0.945
Parent report								
Disease	18	r_s_	0.156	0.010	0.086	0.061	0.060	0.065
		P	0.535	0.969	0.734	0.811	0.815	0.797
Communication	18	r_s_	0.307	−0.351	−0.247	0.078	0.013	0.148
		P	0.216	0.153	0.324	0.760	0.960	0.559
Family	18	r_s_	−0.058	−0.087	0.061	0.004	0.015	−0.028
		P	0.819	0.731	0.811	0.988	0.953	0.913
EQ-5D								
Total mobility	36	r_s_	0.168	−0.150	−0.139	−0.386	−0.344	−0.355
		P	0.328	0.382	0.421	0.020	0.040	0.034
Self-service	37	r_s_	0.092	−0.131	−0.081	−0.219	−0.094	−0.320
		P	0.589	0.439	0.635	0.192	0.580	0.054
Usual activities	37	r_s_	0.135	−0.196	−0.146	−0.278	−0.173	−0.342
		P	0.427	0.245	0.390	0.096	0.305	0.038
Pain	36	r_s_	−0.003	0.238	0.041	0.064	0.233	−0.018
		P	0.987	0.162	0.814	0.709	0.172	0.919
Anxiety	37	r_s_	−0.318	0.330	0.309	−0.235	−0.195	−0.202
		P	0.055	0.046	0.063	0.162	0.248	0.231
Good/bad days	37	r_s_	−0.034	−0.147	−0.026	0.216	0.117	0.221
		P	0.841	0.386	0.878	0.199	0.492	0.188

CMR, cardiac magnetic resonance; EDV, end-diastolic volume; ESV, end-systolic volume; QoL, quality of life; LVEF, left ventricular ejection fraction; MAPSE, mitral annular plane systolic excursion; *p*, *p*-value; PEDSQoL, pediatric quality of life questionnaire.

### PEDSQL 3.0 neuromuscular model questionnaire

**Figure 1:**
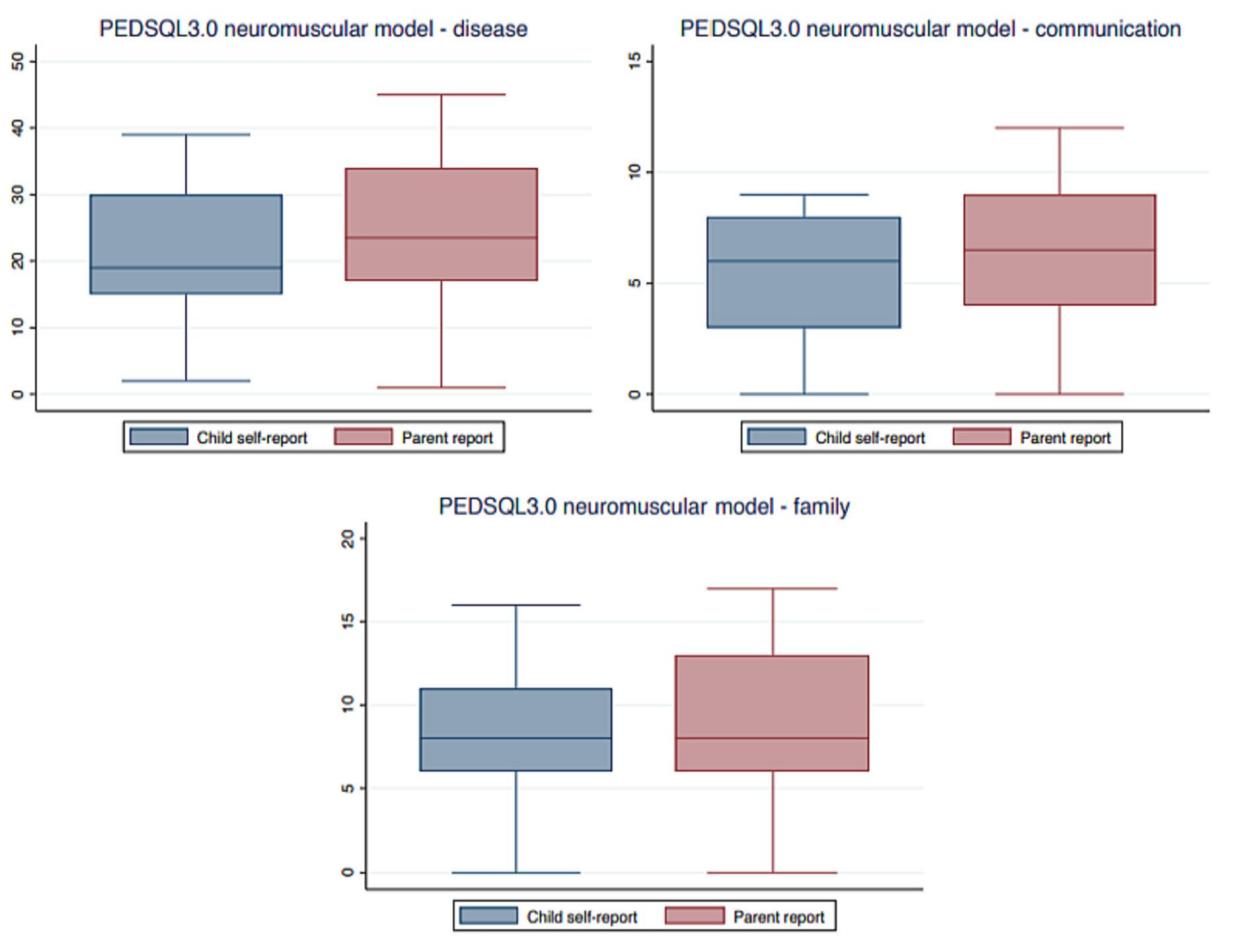
PEDSQL 3.0 neuromuscular model questionnaire completed by DMD patients and their parents. DMD, Duchenne muscular dystrophy; PEDSQL, pediatric quality of life questionnaire.

DMD patients aged 8–18 (median 13.9 years, IQR 11.6–15.6) and their parents completed the PEDSQL 3.0 neuromuscular model questionnaire. A statistically significant differences in disease category was found between the PEDSQL 3.0 neuromuscular model questionnaires completed by the DMD patients and their parents (*p* = 0.008) (see Figure 1).

There was no significant difference in the PEDSQL 3.0 neuromuscular model questionnaire for DMD patients taking or not taking ACEI/sartans, nor for those taking or not taking corticosteroids.

BMI did not correlate with any of the evaluated parameters in the PEDSQL 3.0 neuromuscular model questionnaire.

QoL negatively correlated with movement of the upper limbs using PEDSQL 3.0 neuromuscular model questionnaire in the disease category completed by the DMD patients (r_s_ = −0.748, *p* = 0.0002) and by the parents (r_s_ = −0.742, *p* = 0.0004).

Movement of upper limbs positively correlated with septal MAPSE (r_s_ = 0.3422, *p* = 0.038).

## Discussion

This was the first prospective study describing a detailed evaluation of the QoL in DMD patients of various ages and degrees of disability in relation to functional neurological and cardiac impairment. The most important results of our prospective study were the relation/association between:

1. Better movement of the upper limbs and total mobility, self-service, and good/bad days, but not usual activities.
2. Decreased QoL and movement of the upper limbs using the PEDSQL 3.0 neuromuscular model questionnaire in disease category completed both by DMD patients and their parents.
3. Movement of the upper limbs and septal MAPSE also trended toward average MAPSE.
4. Scoliosis and good/bad days.
5. EDV and anxiety/depression, but not average MAPSE and overall mobility or lateral MAPSE and total mobility.
6. Septal MAPSE and total mobility or usual activities.
7. DMD patients taking ACEI/sartans had better overall mobility than DMD patients without therapy for heart failure.

Compared to the previously published studies focused on QoL in DMD patients (7, 8, 10, 11, 13–25, 27, 29, 37–39), we included all-aged DMD patients, we used the common questionnaires such as EQ-5D and PEDSQL 3.0 neuromuscular model questionnaire, and the results were not under/overestimated due to proxy reports as the PEDSQL 3.0 neuromuscular model questionnaire was completed both by DMD patients and their parents in our study. Possible reasons why DMD patients perceive their QoL as higher compared to their parents include fears about their child’s disorder, the adaptation of DMD patients to their disease, an inexact perception of a child’s status by their parents, limitations of the child, and the influence of environmental factors (13–15, 40). The results of our study were consistent with the previous studies, as the PEDSQL 3.0 neuromuscular model questionnaire completed by the parents showed more decreased QoL of DMD patients in the disease category compared to those who self-reported.

According to Powell et al., a comprehensive and reliable PROM of QoL including physical and social domain (41, 42) that is typically used for DMD patients (43–47) has a significant positive correlation with the PEDSQL 3.0 neuromuscular model questionnaire and the EQ-5D questionnaires used in our study (46). Moreover, we analyzed the most functional parameters related to the QoL for DMD patients that have ever been published.

### Respiratory and cardiac impairments

When assessing the respiratory functions and QoL, the aspects of daily living and disability were highlighted in DMD patients on NIPPV compared to DMD patients without NIPPV. Regardless of the reduction in pulmonary function and daily living activities, DMD patients on NIPPV showed similar HRQoL to patients without NIPPV (27). The relationship between the physical and mental domains contrary to forced vital capacity did not show any statistically significant results (27). It is consistent with cardiomyopathy where DMD patients with or without cardiomyopathy reported similar disability scores (27). In our investigation, a minority of DMD patients necessitated nocturnal NIPPV; hence, we refrained from correlating its influence with the evaluated parameters.

In our study, we focused on cardiac function using CMR and QoL in DMD patients. Based on our previous study, Panovský et al. (48), DMD patients had impaired LV systolic function measured by MAPSE and global LV strain regardless of normal LVEF and the absence of LGE (48). The higher the EDV the higher anxiety/depression in DMD patients, whereas average MAPSE and total mobility or lateral MAPSE and total mobility were associated negatively. Similarly, decreasing septal MAPSE introduced reduced total mobility and usual activities. The better movement of the upper limbs the higher the septal MAPSE value. No arrhythmia was detected based on ECG Holter monitoring in our cohort. When evaluating the presence or absence of LGE and LVEF, there were no statistically significant differences observed in relation to any of the assessed parameters.

According to Porcher et al., DMD patients who take ACEI therapy prophylactically had significantly higher overall survival and lower rates of hospitalization for heart failure (49). This correlates with the results of our study that found DMD patients on ACEI/sartans had better overall mobility than DMD patients without therapy. Contrary to it, there was no significant difference for those taking ACEI/sartans according to the PEDSQL 3.0 neuromuscular model questionnaire.

### Corticosteroid therapy

It is well-known that corticosteroid therapy can improve physical functioning and prolong estimated life expectancy and HRQoL in muscular dystrophy patients (11, 39, 50). We concentrated on influence of current corticosteroid therapy in our cohort. The average age at which corticosteroid therapy was initiated in our cohort is 5 years. All patients receive prednisone at a dose of 0.75 mg/kg/day, with the exception of one patient who takes deflazacort at a dose of 0.9 mg/kg/day. Some patients chose not to undergo corticosteroid therapy due to the gradual progression of the disease or concerns about potential side effects. Additionally, DMD patients discontinued corticosteroid therapy when they were no longer ambulatory, despite some guidelines recommending its use for preventing heart or respiratory failure.

Concerning the QoL, DMD patients taking or not taking corticosteroid therapy did not show any differences in their PEDSQL 3.0 neuromuscular model questionnaire.

### BMI

It was well-recognized that the administration of steroids can result in weight gain and short stature (51). In the case of our study, BMI did not correlate with any of the evaluated parameters in the PEDSQL 3.0 neuromuscular model questionnaire.

### Pain

DMD patients usually do not complain of pain although their ability to manage pain is limited (52, 53). It was not the subject of our study.

### Movement of upper limbs + total mobility + tiredness

Overall mobility in DMD is a crucial item of QoL (46). Younger DMD patients are usually more tired due to the extra work/effort required for movement, whereas older DMD patients move less (46). Focusing on the movement of the upper limbs, we found a positive association with total mobility, self-service, and good/bad days in DMD patients, but it correlated negatively with usual activities. QoL negatively correlated with movement of the upper limbs using the PEDSQL 3.0 neuromuscular model questionnaire in disease category completed both by the DMD patients and their parents. Moreover, movement of the upper limbs positively correlated with septal MAPSE.

When assessing the ambulatory/non-ambulatory and sitting/non-sitting DMD patients, statistically significant difference in total mobility was found. Ambulatory/non-ambulatory and sitting/non-sitting DMD patients showed significant differences in self-service, usual activities, and good/bad days.

DMD patients with/without scoliosis showed a statistically significant difference in good/bad days.

### Anxiety/sadness/mood/good or bad days

The QoL focused on physical activities, health, and friends in DMD patients was reduced (13). Pangalila et al. reported that social issues of QoL were affected/impaired in DMD patients compared to the control group (54). Based on previous studies, it is known that the general mood and feelings assessed for parents were decreased than that for DMD patients (11, 55). From the children’s perspective, physical activities and health and friends were lower (13).

This is consistent with the results in our study in which EDV was positively associated with anxiety/depression, but was negatively associated with average MAPSE and total mobility, as were lateral MAPSE and total mobility.

### Limitations of the study

It was a small sample because of the rare occurrence of DMD and it was a single-centre study. The questionnaire was completed in electronic version. The results of our study cannot be generalized to the worldwide population due to various levels of health care and social possibilities (56–58). In our study, our emphasis was on categorizing DMD patients into two primary groups: ambulatory and non-ambulatory. Specifically, detailed information on five subcategories of upper limb movement was collected exclusively from the “non-ambulatory DMD patients,” with the corresponding data absent for ambulatory DMD patients. Regarding the evaluation of corticosteroid therapy’s influence, our focus was solely on the ongoing pharmacotherapy. Future studies focused on QoL in DMD are needed to confirm our results.

## Conclusion

This study delved into various dimensions of QoL in DMD patients, considering neurological, cardiac, therapeutic, and emotional aspects. Key findings revealed significant correlations: improved upper limb movement positively related to overall mobility and emotional well-being, while QoL displayed negative associations with upper limb movement. Cardiac parameters, especially septal MAPSE, were interlinked with motor function. QoL distinctions were apparent among ambulatory/non-ambulatory and sitting/non-sitting DMD patients. Anxiety/depression correlated with cardiac parameters, highlighting the intricate connection between emotional and cardiac well-being. Treatment with ACEI/sartans positively impacted overall mobility. Despite study limitations, these insights underscore the imperative for personalized care strategies in DMD patient management.

## Data availability statement

The original contributions presented in the study are included in the article/supplementary material, further inquiries can be directed to the corresponding author.

## Ethics statement

The studies involving humans were approved by Institutional Ethics Committee (University Hospital Brno, reference number 20130410-03). The studies were conducted in accordance with the local legislation and institutional requirements. Written informed consent for participation in this study was provided by the participants’ legal guardians/next of kin.

## Author contributions

LJ: Writing – original draft, Writing – review & editing, Conceptualization, Data curation, Formal analysis, Funding acquisition, Investigation, Methodology, Project administration, Resources, Software, Supervision, Validation, Visualization. LM: Writing – original draft, Writing – review & editing, Conceptualization, Data curation, Formal analysis, Funding acquisition, Investigation, Methodology, Project administration, Resources, Software, Supervision, Validation, Visualization. RP: Conceptualization, Data curation, Formal analysis, Funding acquisition, Investigation, Methodology, Project administration, Resources, Software, Supervision, Validation, Visualization, Writing – review & editing. MP: Conceptualization, Data curation, Formal analysis, Funding acquisition, Investigation, Methodology, Project administration, Resources, Software, Supervision, Validation, Visualization, Writing – review & editing. KR: Conceptualization, Data curation, Formal analysis, Funding acquisition, Investigation, Methodology, Project administration, Resources, Software, Supervision, Validation, Visualization, Writing – review & editing. OV: Conceptualization, Data curation, Formal analysis, Funding acquisition, Investigation, Methodology, Project administration, Resources, Software, Supervision, Validation, Visualization, Writing – review & editing. VF: Conceptualization, Data curation, Formal analysis, Funding acquisition, Investigation, Methodology, Project administration, Resources, Software, Supervision, Validation, Visualization, Writing – review & editing. TH: Conceptualization, Data curation, Formal analysis, Funding acquisition, Investigation, Methodology, Project administration, Resources, Software, Supervision, Validation, Visualization, Writing – review & editing. VK: Conceptualization, Data curation, Formal analysis, Funding acquisition, Investigation, Methodology, Project administration, Resources, Software, Supervision, Validation, Visualization, Writing – review & editing. PD: Conceptualization, Data curation, Formal analysis, Funding acquisition, Investigation, Methodology, Project administration, Resources, Software, Supervision, Validation, Visualization, Writing – review & editing. SV: Conceptualization, Data curation, Formal analysis, Funding acquisition, Investigation, Methodology, Project administration, Resources, Software, Supervision, Validation, Visualization, Writing – review & editing. JH: Conceptualization, Data curation, Formal analysis, Funding acquisition, Investigation, Methodology, Project administration, Resources, Software, Supervision, Validation, Visualization, Writing – review & editing. KP: Conceptualization, Data curation, Formal analysis, Funding acquisition, Investigation, Methodology, Project administration, Resources, Software, Supervision, Validation, Visualization, Writing – review & editing.

## Glossary

**Table:** 

ACEI	Angiotensin converting enzyme inhibitors
BMI	Body mass index
CMR	Cardiac magnetic resonance
DMD	Duchenne muscular dystrophy
ECG	Electrocardiogram
EDV	End-diastolic volume
EF	Ejection fraction
ESV	End-systolic volume
HRQoL	Health-related quality of life
IQRs	Interquartile ranges
LGE	Late gadolium enhancement
LV	Left ventricular
LVEF	Left ventricular ejection fraction
MAPSE	Mitral annular plane systolic excursion
NIPPV	Non-invasive positive pressure ventilation
PEDSQL	Pediatric quality of life questionnaire
PROM	Patient-reported outcome measure
QoL	Quality of life
SDs	Standard deviation

## Footnotes

^1^ mapi-trust.org
